# Molecular Characterization of Pathogenic Members of the Genus *Fonsecaea* Using Multilocus Analysis

**DOI:** 10.1371/journal.pone.0041512

**Published:** 2012-08-02

**Authors:** Jiufeng Sun, Mohammed J. Najafzadeh, Albertus H. G. Gerrits van den Ende, Vania A. Vicente, Peiying Feng, Liyan Xi, Gerrit S. De Hoog

**Affiliations:** 1 Department of Dermatology, Sun Yat-sen Memorial Hospital, Sun Yat-sen University, Guangzhou, Guangdong, China; 2 Department of Parasitology, Zhongshan School of Medicine, Key Laboratory for Tropical Disease Control, Ministry of Education, Sun Yat-sen University, Guangzhou, Guangdong, China; 3 CBS-KNAW Fungal Biodiversity Centre, Utrecht, The Netherlands; 4 Institute for Biodiversity and Ecosystem Dynamics, University of Amsterdam, The Netherlands; 5 Department of Parasitology and Mycology, and Cancer Molecular Pathology Research Center, Ghaem Hospital, School of Medicine, Mashhad University of Medical Sciences, Mashhad, Iran; 6 Department of Basic Pathology, Federal University of Paraná, Curitiba, PR, Brazil; 7 Department of Dermatology, Third Affiliated Hospital, Sun Yat-sen University, Guangzhou, Guangdong, China; 8 Peking University Health Science Center, Research Center for Medical Mycology, Beijing, China; New York State Health Department and University at Albany, United States of America

## Abstract

Members of the fungal genus *Fonsecaea* causing human chromoblastomycosis show substantial geographic structuring. Genetic identity of clinical and environmental strains suggests transmission from plant debris, while the evolutionary processes that have led to spatially separated populations have remained unexplained. Sequences of ITS, *BT2*, *ACT1*, *Cdc*42, *Lac* and *HmgA* were analyzed, either by direct sequencing or by cloning. Thirty-seven clinical and environmental *Fonsecaea* strains from Central and South America, Asia, Africa and Europe were sequenced and possible recombination events were calculated. Phylogenetic trees of *Cdc*42, *Lac* and *HmgA* were statistically supported, but ITS, *BT2* and *ACT1* trees were not. The Standardized Index of Association (I_A_^S^) did not detect recombination (I_A_^S^ = 0.4778), neither did the *Phi*-test for separate genes. In *Fonsecaea nubica* non-synonymous mutations causing functional changes were observed in *Lac* gene, even though no selection pressures were detected with the neutrality test (Tajima D test, *p*>0.05). Genetic differentiation of populations for each gene showed separation of American, African and Asian populations. Strains of clinical *vs.* environmental origin showed genetic distances that were comparable or lower than found in geographic differentiation. In conclusion, here we demonstrated clonality of sibling species using multilocus data, geographic structuring of populations, and a low functional and structural selective constraint during evolution of the genus *Fonsecaea*.

## Introduction

The genus *Fonsecaea* comprises etiologic agents of human chromoblastomycosis, a chronic (sub)cutaneous infection eventually leading to cauliflower-like eruptions on the skin [1], [2]. The fungus is present in human tissue in the form of muriform cells. The disease has been reported worldwide, but mostly in tropical and subtropical climate zones, with high incidence in endemic areas [3]–[7].

Inoculation of contaminated thorns or wooden splinters has been hypothesized to be a main route of infection [8], [9]. Thus far the etiologic agents within *Fonsecaea* are limited to three closely related siblings composing a clearly delimited clade [10]: *Fonsecaea pedrosoi*, *F. monophora* and *F. nubica*. Environmental sampling to recover the species from their supposed natural habitat has been done [8], [9]. *F. pedrosoi* and *F. monophora* were only rarely encountered. However, the majority of *Fonsecaea*-like strains concerned non-virulent species, which were not frequently isolated from on human infections [8]. Either the natural habitat of pathogenic *Fonsecaea* species has to be found somewhere else, or, alternatively, the species have some kind of advantage of being carried by a mammal host. The existence of evolutionary processes supporting the latter hypothesis may be revealed by comparing patterns of variability and distribution of potential etiologic agents.

The pathogenic strains form a well-supported clade in the Chaetothyriales [11], but specific delimitation within this clade is still a debated issue. Analysis of global genetic diversity using AFLP showed that five groups were distinguishable, which were considered to belong to three different species. *Fonsecaea pedrosoi* was relatively homogeneous and was found nearly exclusively in Central and South America, while *F. monophora* and *F. nubica* each comprised several AFLP groups and had worldwide distribution. Cases were found in a tropic climate zone around the equator, while the few clinical cases outside endemic areas were supposed to have been distributed by recent migration of the human host [11].

In the present study, we investigate patterns of variability of pathogenic *Fonsecaea* species using multilocus analysis of five functional genes with anonymous sequence and AFLP markers. The set of strains analyzed comprised clinical and environmental strains from three continents.

## Materials and Methods

### Ethical Standards

The present study has been fully reviewed and approved by Sun Yat-Sen University’s Academic Committee. All subjects provided written informed consent and the procedures have been approved by the Sun Yat-sen University Medical Ethics Committee.

### Fungal Strains and Culture Conditions

Seventeen strains of *F. pedrosoi*, 12 of *F. monophora*, 8 of *F. nubica* (Table 1) and one of a neighbouring *Cladophialophora* species were obtained from the reference collection of the Centraalbureau voor Schimmelcultures Fungal Biodiversity Centre (Utrecht, the Netherlands), in addition to fresh strains recovered from patients, and environmental isolates. Stock cultures were maintained on slants of 2% malt extract agar (MEA) and oatmeal agar (OA) at 24°C.

**Table 1:** Detailed information of *Fonsecaea* isolates used in this study.

Taxonomicname	CBS number	origin	Host/sex	Location	AFLPgenotyping	Multilocusgenotyping		
						*Cdc42*	*Lac*	*HmgA*
*F.nubica*	CBS 121733	Chromoblastomycosis	Human/M	China, Guangdong	A	A	A	A
	CBS 121720	Chromoblastomycosis	Human/M	China, Guangdong	A	A	A	A
	CBS 121734	Chromoblastomycosis	Human/M	China, Guangdong	A	A	A	A
	CBS 269.64	Chromoblastomycosis	Human/F	South Africa	ND	B	B	B
	CBS 444.62	Chromoblastomycosis	Human/M	Surinam	ND	B	B	B
	CBS 557.76	Unknown	Unknown	Unknown	B	B	B	B
	CBS 270.37	Unknown	Unknown	France (fromS. America)	B	B	B	B
	CBS 277.29	Chromoblastomycosis	Human/M	Brazil	B	B	B	B
*F.monophora*	CBS 102243	Chromoblastomycosis	Human/M	Brazil, Parana,Ibituva	C	C	C	C
	CBS 117236	Brain	Human/M	United States	C	C	C	C
	CBS 102246	Chromoblastomycosis	Human/M	Brazil, Parana,Campo Largo	C	C	C	C
	CBS 269.37	Chromoblastomycosis	Human	South America	C	C	C	C
	CBS 102238	Soil	Soil	Brazil, Parana,Tibagi River	C	C	C	C
	CBS 102229	Decaying vegetable cover	Plant	Brazil, Parana,Piraquara	C	C	C	C
	CBS 397.48	Chromoblastomycosis	Human/M	South America	C	C	C	C
	CBS 102248	Chromoblastomycosis	Human/M	Brazil, Parana,Piraquara	C	C	C	C
	CBS 121727	Chromoblastomycosis	Human/M	China, Guangdong	D	D	D	C
	CBS 121721	Chromoblastomycosis	Human/M	China, Guangdong	D	D	D	C
	CBS 117238	Brain	Human	United Kingdom	D	D	D	C
	CBS 121724	Chromoblastomycosis	Human/M	China, Guangdong	D	D	D	C
*F.pedrosoi*	CBS 273.66	Mouse passage	Soil	Venezuela	ND	E	E	D
	CBS 271.37	Chromoblastomycosis	Human/M	South America	E	E	E	D
	CBS 671.66	Mouse passage	Soil	Venezuela	E	E	E	D
	CBS 274.66	Mouse passage	Soil	Venezuela	E	E	E	D
	CBS 102247	Chromoblastomycosis	Human/M	Brazil, Parana	E	E	E	D
	CBS 122740	Chromoblastomycosis	Human/M	Mexico, Mexico City	E	E	E	D
	CBS 122736	Chromoblastomycosis	Human/M	Mexico, Mexico City	E	E	E	D
	CBS 122849	Chromoblastomycosis	Human/M	Mexico, Mexico City	E	E	E	D
	CBS 285.47	Chromoblastomycosis	Human/M	Puerto Rico	E	E	E	D
	CBS 342.34	Chromoblastomycosis	Human/M	Puerto Rico	E	E	E	D
	CBS 122741	Chromoblastomycosis	Human/M	Mexico, Mexico City	E	E	E	D
	CBS 670.66	Mouse passage	Soil	Venezuela	E	E	E	D
	CBS 212.77	Chromoblastomycosis	Human/M	Netherlands,Amsterdam	E	E	E	D
	CBS 117910	Chromoblastomycosis	Human/M	Venezuela, Coro,Falcón State	E	E	E	D
	CBS 272.37	Chromoblastomycosis	Human	Brazil	E	E	E	D
	CBS 253.49	Chromoblastomycosis	Human	Uruguay,Montevideo	E	E	E	D
	CBS 201.31	Gazelle, ear	Animal	Libya, Cyrenaica,Derna	E	E	E	D
*Cladophialophora* sp.	CBS 109631	Unknown	Human	Uruguay	F	F	F	E

CBS: Centraalbureau voor Schimmelcultures Fungal Biodiversity Centre, Utrecht, Netherlands.

ND: not determined.

### DNA Extraction and Identification

DNA extraction and quality test were performed as previously reported [12], [13]. DNA concentrations were measured with nano-drop DNA concentration detector at 260 nm (Thermo Scientific, U.S.A.).

### Degenerate Primer Design, Cloning and Specific Primer Design for Cdc42, Lac and HmgA

Degenerate and specific primers of *Cdc42* refer to the study of Xie *et al.* [14]. The degenerate primers of *HmgA* and *Lac* were designed using a complete alignment of the amino acid sequences of species listed in Table 2. Multiple sequence alignments were generated with the software Clustal W [15] using the amino acid substitution matrix BLOSUM62 [16], [17]. Highly conserved areas were chosen for degenerate primer design. Degenerate forward and reverse primers were designed with minimal degenerate degree using Primer 5.0 software (Table 2).

**Table 2:** Degenerate primers and specific primers used in this study.

Degenerate primers				
Gene	primer	Amino acid sequences	Degenerate nucleotide sequences	
*Cdc42*	*Cdc42-F*	G K T C L L I S	GGR AAR ACM TGY YTN ATH TCN TC	
	*Cdc42-R*	L K D V F D E A	GCC TCR TCR AAR ACW KYC TTS A	
*Lac*	*Lac-Ds*	V T H C P I P	GTK ACD CAR TGY CCS ATT CC	
	*Lac-Das*	H G H V H P P	TG SCC RTG VAR RTG GAA CGG	
*HmgA*	*HmgA-F2*	F T A P R H E	TTY ACN GCN CCN MGN CAY GA	
	*HmgA-R12*	N H G N Y Y P	GG RTA RTA RTT NCC RTG CC	
	*HmgA-R22*	P P N Y H R N	TT NCK RTG RTA CCA NGG NGG	
**Specific primers**				
**Gene**	**primer**	**Specific nucleotide sequences**		**Reference**
*Cdc42*	*Cdc42-SF1s*	GGC AAG ACA TGC TTG TTG ATC TC		This study
	*Cdc42-SR1s*	GCC TCG TCA AAT ACG TCC TTA A		
*Lac*	*LacIs*	CGC CAG GCT TTG ATT GTG		This study
	*LacIas*	CGC CGT CGT TAT TGT TGA G		
*HmgA*	*HmgA-F2s*	TTR ACT GCG CCA CGR CAC GA		This study
	*HmgA-R12s*	GG RTA RTA RTT GCC RTG CCA T		
*ITS*	*V9G, LS266*			Masclaux *et al.* (1995)
*BT2*	*Bt2a, Bt2b*			White *et al*. (1990
*ACT1*	*Actaw, Actfw*			Glass & Donaldson (1995)

DNA of type strains of the genus *Fonsecaea* were used as the PCR amplification template. Optimal amplification condition was optimized by temperature gradient PCR amplification. Specific amplicons were purified using gel extraction kit (Qiagen, Germany), cloned using a cloning kit (Promega, Madison, WI, U.S.A.) and confirmed by direct PCR amplification with the primer set M13fw (5′-GTA AAA CGA CGG CCA GT-3′) and M13rv (5′-GGA AAC AGC TAT GAC CAT G-3′) according to the manufacturer’s instructions. PCR amplicons were then purified with Sephadex G-50 fine (GE Healthcare Bio-Sciences, Uppsala, Sweden) and sequencing was done on an ABI 3730XL automatic sequencer (Applied Biosystems, Foster City, CA, U.S.A.). Sequence data were edited using the SeqMan of Lasergene software (DNAStar, Madison, WI, U.S.A.). The resulting sequences were aligned using BioNumerics software v. 4.61 (Applied Maths, Kortrijk, Belgium). The specificity of these sequences for three genes was confirmed by BLASTx search on GenBank (http://blast.ncbi.nlm.nih.gov). Specific primers for *HmgA* and *Lac* were obtained by comparison with the degenerate primer of *HmgA* and *Lac* respectively. The resulting specific primers *cdc42-SF1s*, *cdc42-SR1s*, *Lac-Is, Lac-IAs, HmgA-F2s* and *HmgA-R12s* (Table 2) were subsequently tested with the aim to establish amplification conditions, and then were used to test the 38 strains listed in Table 1.

### Multilocus Gene Amplification and Sequencing

PCR amplification and sequencing of ITS, *BT2, ACT1* was done according our earlier study [18]. PCR amplification of *Cdc*42, *Lac* and *HmgA* was performed with *cdc42-SF1s* and *cdc42-SR1s*, *LacIS* and *LacIAS*, and *HmgA-F2s*, *HmgA-R12s* and *HmgA-R22s*, respectively. PCR was performed in a 50 µl volume of a reaction mixture containing 14 µl Go Taq master mix (Promega) containing dNTPs, MgCl_2_, reaction buffer, 2 µl of each primer (10 pmol) and 1 µl DNA. Amplification was performed in an ABI PRISM 2720 (Applied Biosystems) thermocycler as follows: 95°C for 5 min, followed by 35 cycles consisting of 95°C for 45 sec, 49.5°C for 30 sec and 72°C for 1.5 min, and a delay at 72°C for 7 min. Annealing temperature was changed to 52°C for *Lac*. A seminested PCR was performed to amplify the *HmgA* gene, the first run with primer *HmgA-F2s* and *HmgA-R22,* as follows: 95°C for 5 min, followed by 35 cycles consisting of 95°C for 45 sec, 49.5°C for 30 sec and 72°C for 1.5 min, and a delay at 72°C for 7 min. One µl amplicon of the first run were used as templates for the second run with primer *HmgA-F2s* and *HmgA-R12s* under the same reaction conditions. Sequencing of PCR amplicons was done on an ABI 3730XL automatic sequencer (Applied Biosystems, U.S.A.). Sequence data were edited using the SeqMan of Lasergene software (DNAStar Inc., Madison, U.S.A.).

### Phylogenetic Reconstruction and DNA Polymorphism

The Cipres Portal (http://www.phylo.org) was used to construct maximum likelihood trees with RAxML v. 7.2.6 for ITS, *BT2*, *ACT1*, *Cdc*42, *Lac* and *HmgA*. Maximum likelihood searches for the best scoring tree were made after a bootstrap estimate of the proportion of invariable sites automatically determined the number of bootstrapping runs. RAxML will then automatically determine the point at which enough bootstrapping replicates have been produced [19]. Bootstrap values equal to or greater than 80% were considered significant. After repeated construction for all six markers, the combined single file was used to calculate the standardized Index of Association, I_A_^S^ [20] using the Lian 3.5 webserver (http://pubmlst.org). The test options were set to Monte Carlo with 1,000 iterations/random resamplings. The same alignments were used to show split-decomposition trees using Splitstree 4 v. 4.8. The same software package was used to apply *Phi* test (pairwise homoplasy index) to distinguish recurrent mutations (or homoplasies) from recombination in generating genotypic diversity.

DNA polymorphism analyses were carried out using D_NA_SP 5.10.00 software. A subset of *Fonsecaea* strains and genotypes was used to calculate haplotype and nucleotide diversity, as well as Tajima’s *D* neutrality test that is based on the number of pairwise differences and the number of segregating sites in a sample of sequences and the number of parsimonious informative sites [21]. The same software package was used to calculate *F_ST_*, showing the genetic differentiation among populations, for ITS, *BT2*, *ACT1*, *Cdc*42, *Lac* and *HmgA*.

### AFLP Genotyping Assay

AFLP genotyping data were taken from our previous study, where a detailed description of the methodology is provided [11].

### Laccase and Homogentisate 1,2-dioxygenase Enzyme Activity Assays

All strains representing *F. pedrosoi*, *F. monophora* and *F.nubica* indicated in Table 1 were tested for laccase and homogentisate 1, 2-dioxygenase enzyme activities. Tests were repeated three times for each strain. Laccase was tested according to Mander *et al*. [22]. Solid MM with a pH of 5 supplemented with 5 mM 2, 2-azino-di-(3-ethylbenzthiazolinsulfonate) (ABTS) which is oxidized by laccase and results in colored compounds. Cultures were pre-incubated at 25°C for 7 days. Subcultures were cut with a cork borer 2 mm diam and placed at the centre of the plate with three replicates. Diameters of colored metabolite halos were measured from day 1 to day 7. For the homogentisate 1,2-dioxygenase enzyme activity test, we followed Ye & Szaniszlo [23]. Solid MM was supplemented separately with 5 mM L-phenylalanine (Sigma, U.S.A.) and 5 mM L-tyrosine (Sigma) which served as artificial substrates to evaluate the homogentisate 1,2-dioxygenase enzyme activity. Culture conditions were the same as in the laccase test. After two weeks of culture, colony diameters were measured.

### Statistics

Metabolite diameters were analyzed by one way ANOVA using Prism 5.0 software, followed by Tukey’s HSD Post-hoc test. Mean diameters are the result of triplicate experiments. The error bars indicate standard error of the mean; *p*<0.05 was considered to indicate a significant difference.

## Results

### Primer Development for Cdc42, Lac and HmgA

Highly conserved domains were found in *Cdc42, HmgA* and *Lac* genes after comparison of sequences downloaded from GenBank (Table 3) and these were used for degenerate primer design. PCRs with degenerate primer pairs *Cdc42-F* and *Cdc42- R, Lac-Ds* and *Lac-Das, HmgA-F2* and *HmgA-R12/HmgA-R22* yielded multiple bands. After cloning and alignment analysis, the specific primers *cdc42-SF1s* and *cdc42-SR1s*, *Lac-IS* and *Lac-IAS*, and *HmgA-F2s*, *HmgA-R12s*/*HmgA-R22s* were obtained (Table 2). The sets of specific primers each yielded single PCR products of about 0.85 kb, 1 kb and 0.9 kb, respectively (data not shown). The introns were taken out when used for further analysis. The primer sets proved to amplify all *Fonsecaea* agents of chromoblastomycosis successfully. To establish an outgroup, degenerate primers were used to amplify the target gene, and multiple bands were cloned and sequenced. Blast searches using translated amino acid sequences in GenBank showed that the amplified fragments of *Cdc42, Lac* and *HmgA* had high homology with published target genes [24]–[26]. The conserved domain search revealed that *Cdc42* contains a Ras-like GTPase superfamily (aa_1–120_) which involved a GTP/Mg^2+^ binding site (aa_45_–_100_) and switch I and II regions (aa_20–25_, aa_40–60_) [27]. *Lac* contained a Cu-oxidase superfamily which typically exists in the laccase family [27]. *HmgA* contained the *HgmA* superfamily (aa_1–204_), a hexamer arrangement consisting of a dimer of trimers with which the active site iron ion is coordinated [27].

**Table 3:** Homogentisate 1,2-dioxygenase (*HmgA*) and laccase (*Lac*) references taken from GenBank.

Taxonomic name	Associated strain number	gene	GenBank no. protein
*Ajellomyces dermatitidis*	SLH14081	*HmgA*	XP_002626277.1
*Trichophyton tonsurans*	CBS 112818	*HmgA*	EGD98945
*Coccidioides immitis*	RS	*HmgA*	XP_001247541.**1**
*Paracoccidioides brasiliensis*	Pb03	*HmgA*	EEH17396.**1**
*Trichophyton tonsurans*	CBS 112818	*HmgA*	EGD98945.**1**
*Trichophyton equinum*	CBS 127.97	*HmgA*	EGE07801.**1**
*Aspergillus terreus*	NIH2624	*HmgA*	XP_001218689.**1**
*Aspergillus niger*	CBS 513.88	*HmgA*	XP_001388730.2
*Aspergillus oryzae*	RIB40	*HmgA*	XP_001727215.2
*Trichophyton rubrum*	CBS 118892	*HmgA*	XP_003238076.**1**
*Neurospora crassa*	OR74A	*HmgA*	XP_960461.**1**
*Aspergillus fumigatus*	Af293	*HmgA*	XP_750969.**1**
*Penicillium marneffei*	ATCC 18224	*HmgA*	XP_002150285.1
*Neurospora crassa*		*Lac*	AAA33591.1
*Cryptococcus neoformans var. grubii*		*Lac*	ABI58272.1
*Cryptococcus neoformans var. neoformans*	JEC21	*Lac*	AAW46742.1
*Aspergillus nidulans*	FGSC A4	*Lac*	XP_664239.1
*Aspergillus flavus*	NRRL3357	*Lac*	EED57644.1
*Aspergillus terreus*	NIH2624	*Lac*	EAU34323.1
*Talaromyces stipitatus*	ATCC 10500	*Lac*	EED19078.1
*Ajellomyces dermatitidis*	SLH14081	*Lac*	XP_002629368.1
*Penicillium marneffei*	ATCC 18224	*Lac*	EEA21273.1
*Aspergillus clavatus*	NRRL 1	*Lac*	EAW07265.1
*Aspergillus fumigatus*	Af293	*Lac*	XP_752933.1
*Trichophyton tonsurans*	CBS 112818	*Lac*	EGD95875.1
*Coccidioides immitis*	RS	*Lac*	XP_001239516.1

CBS: Centraalbureau voor Schimmelcultures Fungal Biodiversity Centre, Utrecht, Netherlands.

NIH: The National Institute of Heath, Bethesda, Maryland, USA.

ATCC: American Type Culture Collection, Manassas, VA, USA.

FGSC: The Fungal Genetics Stock Center, Kansas City, Missouri, USA.

NRRL: ARS Culture Collection, Washington DC, USA**.**

### Phylogeny

**Figure 1:**
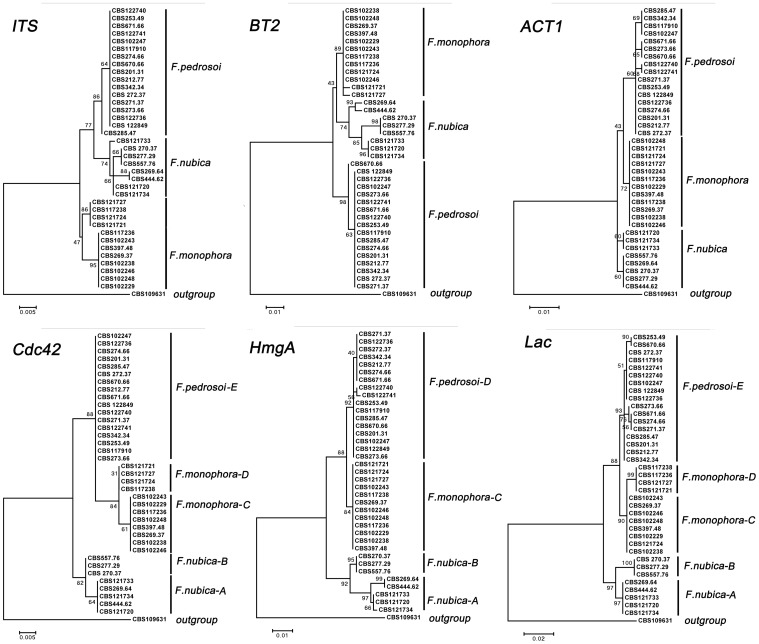
Consensus trees of *Fonsecaea* based on *ITS* ribosomal DNA, *BT2*, *ACT1*, *Cdc42*, *HmgA* and *Lac* of 37 strains, constructed with MEGA5.0 and 500 bootstrap replicates, CBS 109631 was taken as outgroup.

**Figure 2:**
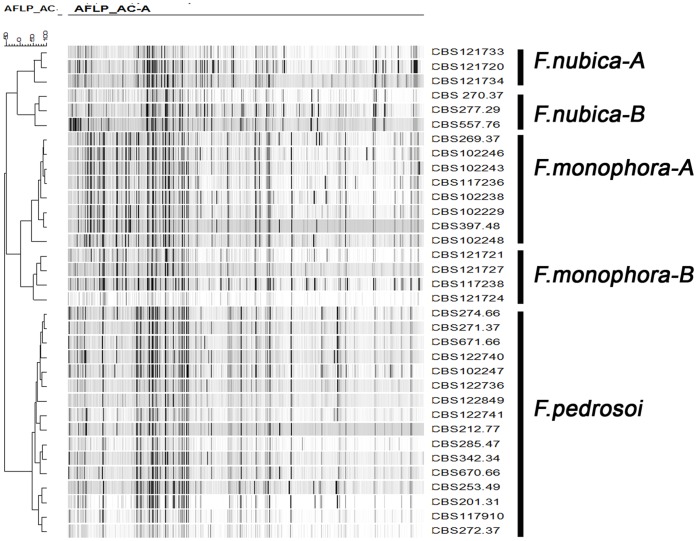
Clustering of amplified fragment-length polymorphism banding pattern of isolates of *Fonsecaea* spp. analyzed by using unweighted pair group method with arithmetic mean. Subclusters showed as in Figure.

Six phylogenetic trees were constructed for 37 *Fonsecaea* strains distributed globally using sequenced ITS, *BT2*, *ACT1*, *Cdc*42, *Lac* and *HmgA* genes, and one *Cladophialophora* strain (CBS 109631) used as outgroup. Three clades corresponding to *F. pedrosoi*, *F. monophora* and *F. nubica* showed strong support in *Cdc*42, *Lac* and *HmgA* genes (bootstrap values >80%) (Fig. 1). *F.pedrosoi* showed limited variability within the species. Two subclades were distinguished within *F. monophora* with high bootstrap support in *Cdc42* and *Lac* (Fig. 1), while within *F.nubica*, two subclades in *Cdc42, Lac* and *HmgA* (Fig. 1). The AFLP genotyping assay showed the similar tree topology (Fig. 2), with five subclades with high bootstrap support within the genus *Fonsecaea.* However, for ITS, *BT2* and *ACT1* genes, no significant bootstrap support was obtained (Fig. 1). Fixed populations were observed throughout the six phylogenetic and AFLP genotyping trees. The *F. pedrosoi* clade comprised 17 strains from patients and from the environment in South America and Europe. *F.monophora* genotype A comprised 4 clinical strains from South China, and genotype B comprised 8 strains from patients and the environment in South America. *F.nubica* genotype A comprised 3 clinical strains from in Europe and South America, and genotype B comprised 5 clinical strains from Africa and China (Table 1, Fig. 1).

### Multilocus Recombination Analyses

Standardized Index of Association I_A_^S^ [28] performed using Lian 3.5 [20] confirmed clades according to the RaxML trees in the different partitions. I_A_^S^ measures the degree of association between alleles at different loci based on the variance in genetic distance between genotypes and is expected to be 0 if populations are freely recombining and >0 if there is an association between alleles. The calculated index using 1000 Monte Carlo resamplings was 0.4778 (*V_D_* = 3.8561, *Ve* = 0.9973), showing no evidence of recombination. The *Phi*-test [29] is performed for individual loci for detection of recombination within sequences with expected recombination events the value is *p*<0.05, otherwise, homoplasy (*p*>0.05) is considered. The results based on six genes showed no significant statistical evidence of recombination at ITS (*p* = 0.076), *BT2* (*p* = 0.112), *ACT1* (*p* = 1.0), *Cdc42* (*p* = 1.0), *Lac* (*p* = 0.79), or *HmgA* (*p* = 0.46).

### Strain Polymorphism Statistics

The calculated parsimonious informative sites, monomorphic sites, segregating sites and the total number of mutations are summarized in Table 4. In total 37 strains were used for all six genes. The haplotype diversity for ITS (0.761), *BT2* (0.743), *ACT1* (0.824), *Cdc42* (0.725), *Lac* (0.883) and *HmgA* (0.820) were in comparable range. For the neutrality test, Tajima’s *D* values for ITS (1.21111), *BT2* (0.27942), *ACT1* (0.65057), *Cdc42* (1.23506), *Lac* (0.63696) and *HmgA* (0.61812) were not statistically supported (*p*>0.10), no positive selection being detected within the tested genus (Table 4).

**Table 4:** Phylogenetic marker diversity and molecular evolutionary parameters for the gene segments examined.

Parameters	Phylogenetic Marker						
	ITS	*Cdc42*	*Lac*	*HmgA*	*BT2*		*ACT1*
Fragment features	Exon/intron	Exon	Exon	Exon	Exon/intron		Exon/intron
No. of sequences	37	37	37	37	37		37
No. of characters	572	360	708	612	303		486
No. of codon	n.a	120	236	204	83		144
	**DNA polymorphism analysis**						
Gaps/missing data	572	360	708	612	278		485
Segregating sites	22	8	37	32	18		10
No. of mutations (η)	22	8	38	32	18		10
No. of haplotypes	6	5	9	7	9		7
Haplotype diversity	0.761	0.725	0.883	0.820	0.743		0.824
Nucleotide diversity	0.01417	0.00662	0.01478	0.01374	0.01682		0.00600
	**Neutrality analysis**						
Tajima’s D test	1.21111 (*p*>0.10)	1.23506 (*p*>0.10)	0.63696 (*p*>0.10)	0.61812 (*p*>0.10)	0.27942 (*p*>0.10)	0.65057(*p*>0.10)	

The values of *F_ST_* lie between 0 (panmictic) and 1 (total separation). The tested *F_ST_* values based on six genes by comparing geographic origins of the strains (Table 5A) show similar values between South and Central America, while those of Chinese and Africa strains were higher. The *F_ST_* values based on six combined genes from clinical (28 strains) and environmental origins (6 strains) (Table 1) showed a comparable or lower value (0.07567) than with comparisons of geographic origins between South America and Central America (0.10549), South America and Africa (0.33106), South America and Asia (0.25447), Central America and Africa (0.41542), Central America and Asia (0.425565), and Asia and Africa (0.17738) (Table 5B). The comparisons of geographic origins between continents low values were found (Table 5) suggesting separation of populations.

**Table 5:** Population differentiation index (*F_ST_*) of 37 *Fonsecaea* strains based on separate (A) and combined (B) multilocus gene sequences clustered by geographical origin. Seven strains from China, 3 from Africa, 17 from South America and 6 from Central America.

A	South America						Central America						Africa					
	*ITS*	*ACT1*	*BT2*	*Cdc42*	*HmgA*	*Lac*	*ITS*	*ACT1*	*BT2*	*Cdc42*	*HmgA*	*Lac*	*ITS*	*ACT1*	*BT2*	*Cdc42*	*HmgA*	*Lac*
**CA**	**0.0865**	**0.1304**	**0.1727**	**0.0768**	**0.0875**	**0.0788**												
**AF**	**0.2658**	**0.2148**	**0.2524**	**0.3874**	**0.2547**	**0.3158**	**0.4755**	**0.3323**	**0.4352**	**0.4640**	**0.3360**	**0.4802**						
**AS**	**0.1890**	**0.2161**	**0.2286**	**0.1638**	**0.1437**	**0.1664**	**0.3989**	**0.5230**	**0.5764**	**0.3121**	**0.3894**	**0.3533**	**0.2341**	**0.2237**	**0.2378**	**0.1536**	**0.0187**	**0.0821**
**B**	**South America**						**Central America**						**Africa**					
**CA**	**0.10549**																	
**AF**	**0.33106**						**0.41542**											
**AS**	**0.25447**						**0.425565**						**0.17738**					

Synonymous and non-synonymous changes of the genus *Fonsecaea* in amino acid sequence in six genes are listed in Table 4. In total 787 amino acid codons were used for the comparison, and 8 1^st^ base, 2 2^nd^ base and 81 3^rd^ base mutations were found within the three species. All 1^st^ base mutations caused non-synonymous changes, but the 2^nd^ base and 3^rd^ base mutations caused synonymous changes. A further analysis showed that the non-synonymous changes in *ACT1* and *BT2* both did not occur in the functional domain (*ACT1*aa_135_, *BT2*aa_81_), while non-synonymous changes in *Lac* and *HmgA* both occurred in functional domains (*Lac*aa_159_, *HmgA*aa_38_, aa_88_, aa_164_, aa_175_). Most non-synonymous changes were observed in *F. nubica,* where all strains isolated to date originate from chromoblastomycosis patients (Table 6).

**Table 6:** Synonymous and non-synonymous changes in DNA and amino acid sequence in *ACT1*, *BT2*, *Cdc42, Lac* and *HmgA* genes of *Fonsecaea spp*.

Gene	Species	Total codon	1^st^ base	2^ed^ base	3^rd^ base	Amino acid change	Strains
*ACT1*	*F. pedrosoi*	144					
	*F. monophora*	144					
	*F. nubica*	144	1		9	CAT→TAT/H→Y	All tested *F. nubica*
*BT2*	*F. pedrosoi*	83	1		3	TAT→GAT/Y→D	CBS 671.66, CBS 273.66, CBS 670.66
	*F. monophora*	83			1		
	*F. nubica*	83			1		
*Cdc42*	*F. pedrosoi*	120					
	*F. monophora*	120			4		
	*F. nubica*	120			4		
*Lac*	*F. pedrosoi*	236	1		5		
	*F. monophora*	236			7		
	*F. nubica*	236	1	1	22	CCG→CTG/P → L	All tested *F. nubica*
*HmgA*	*F. pedrosoi*	204			1		
	*F. monophora*	204			3		
	*F. nubica*	204	4	1	21	AGC→GGC/S→GGCC→ACC/A→TAGC→AAC/S→NGCT→ACT/A→T	All tested *F. nubica*All tested *F. nubica*All tested *F. nubica*All tested *F. nubica*
Total		787	8	2	81		

H: Histidine, Y: Tyrosine, D: Ariginine, P: Proline, L: Leucine, S: Serine, G: Glycine, A: Alanine, T: Threonine, N: Asparagine.

CBS: Centraalbureau voor Schimmelcultures, Utrecht, the Netherlands.

### Laccase and Homogentisate 1,2-dioxygenase Enzyme Activity Assay

**Figure 3:**
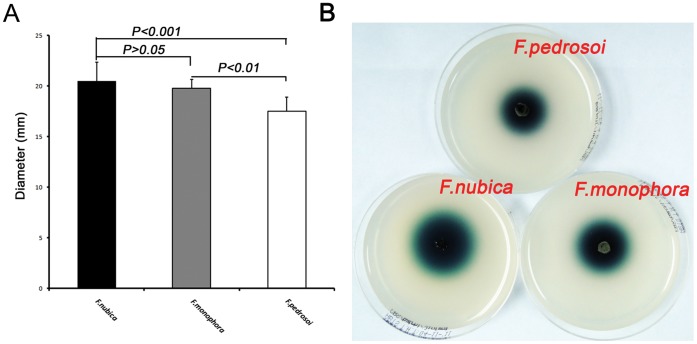
Laccase activity assay. Colored metabolite diameters of tested strains were measured after 7-day culture at 25°C on solid MM medium with 5 mM ABTS. Statistical analysis shown as mean ± standard deviation (A). The plates show the generation of colored metabolite compound (B), *F. pedrosoi* (CBS 273.66), *F. monophora* (CBS 117236), *F. nubica* (CBS 121720).

All strains tested yielded positive laccase activity. Colored metabolites were observed in all three species, but statistical analysis showed that *F. nubica* had higher enzyme activity than other species (*F. nubica* vs. *F. pedrosoi*, *p*<0.001, *F. nubica* vs. *F. monophora*, *p*>0.05, *F. monophora* vs. *F. pedrosoi*, *p*<0.01) (Fig. 3). The homogentisate 1,2-dioxygenase enzyme activity assay revealed that all strains are able to assimilate L-phenylalanine and L-tyrosine as sole carbon sources; no difference was observed within the three species (data not shown).

## Discussion

In the evolution of black fungi (order *Chaetothyriales*) [30], we witness a functional change from a rock-inhabiting life style prevalent in ancestral *Coniosporium (Knufia)* and relatives to an increased ability to infect humans and other vertebrates in derived clades. Agents of chromoblastomycosis are particularly interesting because they exhibit a pathogenic phase in tissue, the muriform cell, which shows morphogenetic resemblance isodiametrically enlarging cell clumps of rock-inhabiting *Coniosporium (Knufia)* species. A functional change in the *Cdc42* gene, involved in cellular polarity has been hypothesized [31]. The change of life style seems to have been quite successful in the *F. pedrosoi* clade, judging from the fact that three related species are nearly exclusively found on humans [32]. Nevertheless the shift was not seen to be reflected in the cytoskeleton-associated *Cdc42* gene when compared over the order *Chaetothyriales* [33].

In the present study, six genes were compared in human-pathogenic *Fonsecaea* species. ITS was used as a standard for phylogenetic construction. *ACT1*, *BT2* and *Cdc42* play a role in cell cycle progression and actin cytoskeleton construction, and are involved in morphogenetic switching, leading to large spherical cells with subsequent cellular division giving rise to the infective muriform cell [34]. *Lac* and *HmgA* are well-documented virulence factors of black fungi, and participate in the synthesis of melanin. DHN melanin is negatively charged, hydrophobic and of high molecular weight, and arises by the oxidative polymerization of phenolic and/or indolic precursors [35]. Melanin enhances virulence in black fungi of the order *Chaetothyriales* [36]–[42]. We developed primers to amplify *Cdc42, Lac* and *HmgA* which proved to be specific for *Fonsecaea*. The sequenced genes were aligned and confirmed to be *Cdc42, Lac* and *HmgA* using BLAST oine search in GenBank. The genes contained the gene-specific conserved domains when searched with translated amino acid sequences [27].

The phylogenetic trees reconstructed with *Cdc42, Lac* and *HmgA* (Fig. 1) yielded high bootstrap support for the three sibling *Fonsecaea* species, while the ITS, *ACT1* and *BT2* trees were not supported. The lack of support was probably caused by incomplete lineage sorting, several mutations not having reached fixation. Based on the Standardized Index of Association (I_A_^S^) and *Phi*-test using six genes, no recombination events were detected among the three sibling species. This phenomenon is frequently observed in opportunistic members of *Chaetothyriales*, where clonality seems to be prevalent [43]. The neutrality test with Tajima’s *D* yielded no significant results, suggesting that no positive selection was detected in the sequenced genes indicating a low functional and structural selective constraint during evolution.

Relatively low haplotype diversity was observed within the six genes analyzed. A total of 91 fixed synonymous and non-synonymous changes were observed in coding regions. The non-synonymous changes in the cytoskeleton genes *ACT1* and *BT2* are not responsible for morphogenetic changes [7], [44] among the three species because the mutations occurred outside functional domains. The non-synonymous changes in *Lac* and *HmgA* both occurred in functional domains (*Lac*aa_159_, *HmgA*aa_38_, aa_88_, aa_164_, and aa_175_) (Table 6), but did not cause obvious functional changes when catalysis of substrates was tested *in vitro*. A possible explanation might be that the non-synonymous mutations did not cause any changes in the three-dimensional structure of the molecule. A systematic alignment of 223 plant and fungi laccase sequences showed that there are four signature sequence regions (L1-4) and 12 housekeeping amino acids [45], while the detected non-synonymous mutations (*Lac*aa_159_) in this study occurred between L2 and L3 and do not belong to a conserved region. DNA sequence alignment of *HmgA* showed that *HmgA*aa_38_, aa_88_, aa_164_, and aa_175_ are not located in conserved regions either. Therefore we conclude that the non-synonymous changes within two genes are not linked to functional or structural selective constraints within the genus *Fonsecaea*. Subsequent studies may reveal whether such changes have occurred in the analyzed genes in ancestral clades, where dramatic changes in life style are supposed to have taken place.

Several studies reported on the molecular epidemiology of the sibling species *Fonsecaea* [45]. Ribosomal and mitochondrial DNA typing has been used to map the geographic origins of strains [46], [47]. The molecular epidemiology of this genus showed substantial geographic structuring in all species with differences between American, African and Asian populations similar to what has been found by Kawasaki et al. [46] in mtDNA profiles. In conclusion, we demonstrated clonality of sibling species using multilocus data, geographic structuring of populations, and a detected low functional and structural selective constraint during evolution of the genus *Fonsecaea*.
