# Correction: Molecular Characterization of Pathogenic Members of the Genus *Fonsecaea* Using Multilocus Analysis

**DOI:** 10.1371/annotation/8fef18d2-c01c-4f66-9b75-ecbfac5c82a5

**Published:** 2012-10-12

**Authors:** Jiufeng Sun, Mohammed J. Najafzadeh, Albertus H. G. Gerrits van den Ende, Vania A. Vicente, Peiying Feng, Liyan Xi, Gerrit S. De Hoog

The second author's name was spelled incorrectly. The correct name is: Mohammad J. Najafzadeh.

